# Group A Streptococcus Subcutaneous Infection-Induced Central Nervous System Inflammation Is Attenuated by Blocking Peripheral TNF

**DOI:** 10.3389/fmicb.2019.00265

**Published:** 2019-02-19

**Authors:** Ya-Hui Liu, Pei-Hua Wu, Chih-Cheng Kang, Yau-Sheng Tsai, Chuan-Kai Chou, Chung-Tiang Liang, Jiunn-Jong Wu, Pei-Jane Tsai

**Affiliations:** ^1^Department of Pathology, National Cheng Kung University Hospital, College of Medicine, National Cheng Kung University, Tainan, Taiwan; ^2^Department of Medical Laboratory Science and Biotechnology, Medical College, National Cheng Kung University, Tainan, Taiwan; ^3^Institute of Clinical Medicine, National Cheng Kung University, Tainan, Taiwan; ^4^Research Center of Clinical Medicine, National Cheng Kung University Hospital, Tainan, Taiwan; ^5^National Laboratory Animal Center, National Applied Research Laboratories, Taipei, Taiwan; ^6^Novo Nordisk Research Centre China, Beijing, China; ^7^Department of Animal Facility, Discovery Biology China, Beijing, China; ^8^Department of Biotechnology and Laboratory Science in Medicine, School of Biomedical Science and Engineering, National Yang-Ming University, Taipei, Taiwan; ^9^Research Center of Infectious Disease and Signaling, National Cheng Kung University, Tainan, Taiwan

**Keywords:** *Streptococcus pyogenes*, TNF, central nervous system inflammation, NF-κB activation, reporter mice

## Abstract

Group A streptococcus (GAS) infection causes a strong inflammatory response associated with cytokine storms, leading to multiorgan failure, which is characterized as streptococcal toxic shock syndrome. However, little is known about GAS subcutaneous infection-mediated brain inflammation. Therefore, we used a bioluminescent GAS strain and reporter mice carrying firefly luciferase under transcriptional control of the nuclear factor-kappa B (NF-κB) promoter to concurrently monitor the host immune response and bacterial burden in a single mouse. Notably, in addition to the subcutaneous inoculation locus at the back of mice, we detected strong luminescence signals from NF-κB activation and increased inflammatory cytokine production in the brain, implying the existence of central nervous system inflammation after GAS subcutaneous infection. The inflamed brain exhibited an increased expression of glial fibrillary acidic protein and nicotinamide adenine dinucleotide phosphate oxidase components and greater microglial activation and blood–brain barrier (BBB) disruption. Furthermore, Fluoro-Jade C positive cells increased in the brain, indicating that neurons underwent degeneration. Peripheral tumor necrosis factor (TNF), which contributes to pathology in brain injury, was elevated in the circulation, and the expression of its receptor was also increased in the inflamed brain. Blockage of peripheral TNF effectively reduced brain inflammation and injury, thereby preventing BBB disruption and improving survival. Our study provides new insights into GAS-induced central nervous system inflammation, such as encephalopathy, which can be attenuated by circulating TNF blockage.

## Introduction

Group A streptococcus (GAS; *Streptococcus pyogenes*) is an important human Gram-positive pathogen responsible for a wide variety of diseases, ranging from mild superficial infections to severe invasive soft tissue infections (Tan et al., 2014; Brouwer et al., 2016). Severe invasive soft tissue infections can cause necrotizing fasciitis and streptococcal toxic shock syndrome (Stevens, 2000; Walker et al., 2014). Although severe streptococcal diseases occur in only a small percentage of cases, they usually lead to systemic inflammation and induce high morbidity and mortality. Reports of GAS invasive diseases have increased worldwide in recent years, and GAS has reemerged as a public health threat (Carapetis et al., 2005; Ralph and Carapetis, 2013). Despite their rare occurrence, brain abnormalities associated with GAS infection have been described. GAS can directly invade the brain tissue or meningeal space causing brain abscess and meningitis (Jagdis, 1988; Paul and Jerwood, 2012). In addition, GAS induces autoantibody production in the condition known as pediatric autoimmune neuropsychiatric disorders associated with streptococci (PANDAS), which involves a possible association with recurrent streptococcal infections (Orefici et al., 2016). Notably, some cases of GAS infection involving brain illnesses without GAS in the brain and not caused by GAS recurrence have been reported (Maytan, 2000; Yanagawa et al., 2009).

Sepsis-associated encephalopathy (SAE) is a brain illness caused by bacterial infection. SAE is one of the common complications of sepsis without direct bacterial infection of the brain. Although many mechanisms for the formation and development of SAE have been proposed, its pathophysiology is still poorly understood. It is considered to entail diffused brain injuries due to blood–brain barrier (BBB) dysfunction, cerebral inflammation, and microglial activation (Gofton and Young, 2012; Adam et al., 2013). Microglial activation plays a major role in inflammatory cytokine and ROS production in the cortex and hippocampus during SAE (Hoogland et al., 2015; Michels et al., 2015). Furthermore, evidence has revealed that tumor necrosis factor (TNF) is involved in the pathogenesis of SAE (Van Gool et al., 2010; Dal-Pizzol et al., 2014). Serum TNF level is increased and TNF could directly act on its receptor, TNFR1, in the brain in lipopolysaccharide-induced encephalopathy (Alexander et al., 2008). However, whether subcutaneous infection can induce central nervous system inflammation is not clear because of inadequate translational studies.

We used the dual reporter system with both bioluminescent GAS and nuclear factor-kappa B (NF-κB) reporter mice to monitor both the location of the bacteria and inflammatory effect in the host. Mice subcutaneously infected with GAS at the back site are susceptible induce central nervous system inflammation, and the effect can be block by inhibition of the peripheral effect of inflammatory cytokines. Subcutaneous infection of GAS-induced central nervous system inflammation is similar to SAE with respect to the occurrence of central nervous system (CNS) histopathology, with an increase in BBB permeability and the induction of inflammation, suggesting that GAS subcutaneous infection can cause brain damage.

## Materials and Methods

### NF-κB Reporter Mice

A 4-kb transgene was separated from pNF-kB-Luc vector (BD Clontech, Palo Alto, CA, United States) and injected into the pronuclei of single cell fertilized embryos isolated from FVB/N mice. The NF-κB-RE (Responsive)-luc transgenic mice were maintained under conditions compliant with the rules and guidelines of the Institutional Animal Care and Use Committee of National Cheng Kung University. Female mice were used for excluding the autoactivation signal of NF-κB from testis.

### Bacterial Strain

*Streptococcus pyogenes* strain A20 (*emm* sequence type 28) was isolated from a blood sample from a patient with necrotizing fasciitis at the National Cheng Kung University Hospital. A20 Tn4001-8, a bioluminescent strain, was produced by J. J. Wu, Department of Medical Technology, National Cheng Kung University. A20 Tn4001-8 is a bioluminescent strain generated from A20 by the transformation with the plasmid pXen-*lux*CDABE (Xenogen Bioware, Alameda, CA, United States) and selected with kanamycin. The bioluminescent phenotype of A20 Tn4001-8 was screened using the xenogeny IVIS 200 (PerkinElmer, Santa Clara, CA, United States). A20 Tn4001-8 was grown in tryptic soy broth supplemented with 0.5% yeast extract and 400 μg/ml kanamycin.

### Subcutaneous Infection and DN-TNF Treatment

The model of subcutaneous infection was modified as previously described (Lu et al., 2013). Female mice at 8–10 weeks of age were anesthetized and hair was then removed from a portion of their backs. An air pouch was produced by subcutaneous injection of 0.2 mL of bacterial suspension (2 × 10^9^ CFU of *S. pyogenes* strain A20 Tn4001-8). Dominant-negative (DN)-TNF (XENP1595) was a soluble selective inhibitor obtained from Xencor, Inc. Three dosages of DN-TNF (50 mg/kg) were intraperitoneally administered 30 min before infection, 3 h post infection, and 6 h post infection.

### Bioluminescence Imaging

Mice were anesthetized and imaged using the xenogeny IVIS 200 (PerkinElmer, Santa Clara, CA, United States). To acquire images of the bacterial luciferase, emission filter wavelengths ranging from 500 to 540 nm were used. To acquire images of firefly luciferase, luciferin (150 mg/kg of animal body weight) was intraperitoneally injected 10 min prior to imaging and images were then acquired using emission filter wavelengths ranging from 620 to 660 nm. To differentiate signals from the bacterial and firefly luciferases, spectral unmixing was performed as previously described (Kadurugamuwa et al., 2005) and analyzed using Living Image software v3.1.

### RNA Isolation and Real-Time PCR

Total RNA was extracted from whole brains using REzol (PROtech, Taipei, Taiwan). Reverse transcription was performed using M-MLV reverse transcriptase (Invitrogen, Carlsbad, CA, United States) and oligo(dT) primers. Real-time PCR was performed using SYBR as implemented in the ABI StepOnePlus Real-Time PCR System (Applied Biosystems, Foster City, CA, United States). Expression of each gene was normalized to β-actin. The sequences of primers were as follows: forward: 5′- CATCTTCTCAAAATTCGAGTGACAA-3′ and reverse: 5′-TGGGAGTAGACAAGGTACAACCC-3′ for TNF; forward: 5′-GCAACTGTTCCTGAACTCAACT-3′ and reverse: 5′-ATCTTTTGGGGTCCGTCAAT-3′ for IL-1β; forward: 5′-CCCACTCACCTGCTGCTACT-3′ and reverse: 5′-TCTGGACCCATTCCTTCTTG-3′ for MCP-1; forward: 5′-GCCATCAGCAACAACATAAGCGTC-3′ and reverse: 5′-CCACTCGGATGAGCTCATTGAATG-3′ for IFN-γ; forward: 5′-GAGAACAACCTGGCTGCGTAT-3′ and reverse: 5′-GCCTCGTATTGAGTGCGAAT-3′ for GFAP; forward: 5′-AGCAGCGGCTCCATGACT-3′ and reverse: 5′-TCATGCGGCCTCCTTTGA-3′ for iNOS; forward: 5′-ACCTGAAACTGCCCACTGAC-3′ and reverse: 5′-ACCTGAAACTGCCCACTGAC-3′ for Ncf1; forward: 5′-GCAGTGGCCTACTTCCAGAG-3′ and reverse: 5′-ACCTCACAGGCAAACAGCTT-3′ for Ncf2; forward: 5′-TTCCTGTTGTCGGTGCCTGC-3′ and reverse: 5′-TTCTTTCGGACCTCTGCGGG-3′ for Cyba; forward: 5′-GGAGTTCCAAGATGCCTGGA-3′ and reverse: 5′-CCACTAACATCACCACCTCATAGC-3′ for Cybb; forward: 5′-CTGTTTGTTCGAGAGCATAAC-3′ and reverse: 5′-TAGGTACTTCTTCATGGCTG-3′ for MPO; forward: 5′-CCTGGAACTCACACGACATCTTC-3′ and reverse: 5′-TGGAAACTCACACGCCAGAA-3′ for MMP-9; forward: 5′-TCAAAGAGGAGAAGGCTGGAAA-3′ and reverse: 5′-CACCACAGCATACAGAATCGCA-3′ for TNFR1; forward: 5′-AAGGGTGGCATCTCTCTTCCA-3′ and reverse: 5′-AGGCACCTTGGCATCTCTTTG-3′ for TNFR2; forward: 5′-ACTGCCGCATCCTCTTCCTC-3′ and reverse: 5′-TGCCACAGGATTCCATACCC-3′ for β-actin.

### BBB Permeability

Sodium fluorescein was used to assess BBB permeability as described previously (Esaki et al., 2010). Mice were intraperitoneally injected with 200 μL of 10% sodium fluorescein (Sigma-Aldrich, St. Louis, MO, United States). After 40 min, the mice were anesthetized and perfused with 10 mL of phosphate-buffered saline (PBS). The brain was then removed and homogenized in 50% trichloroacetic acid and centrifuged at 10,000 g for 10 min. The supernatant was diluted with 0.8 volume of 5 M NaOH and measured using a fluorimeter at an excitation of 485 nm and emission of 515 nm. Sodium fluorescein standard solutions (1–1000 ng/mL) were used to calculate the tissue content, which was normalized to the total amount of protein in the homogenate.

### Serum Cytokines

Serum cytokines were measured using a Milliplex MAP Mouse Cytokine Kit (Millipore, Billerica, MA, United States) on a Luminex 200 analyzer (Luminex, Austin, TX, United States). Data were evaluated by applying a 5-parameter logistic curve fit by using the Software Luminex IS 2.3.

### Iba-1 Immunofluorescence and Fluoro-Jade C Staining

Mice were transcardially perfused with 4% paraformaldehyde (PFA) in PBS. For Iba-1 immunofluorescence, the brain was removed, postfixed in 4% PFA for 24 h, and then transferred to 30% sucrose for 24 h. Cryostat brain sections (10-μm-thick) were fixed in ethanol, blocked with normal serum, and incubated overnight at 4°C with primary antibodies against Iba-1 (1:1000, Dako, Carpinteria, CA, United States), followed by secondary antibodies (Alexa Fluor^®^ 488, Life Technologies, Grand Island, NY, United States). Cell nuclei were counterstained with Hoechst. Images were visualized using a confocal microscope and processed using the EZ-C1 software (Nikon, Tokyo, Japan). For Fluoro-Jade C staining, the deparaffinized and rehydrated slides were transferred to 0.06% potassium permanganate solution for 10 min. After 1–2 min of water wash, the slides were transferred to a solution of 0.0001% Fluoro-Jade C dissolved in 0.1% acetic acid for 10 min. The slides were washed with distilled water, coverslipped, and subsequently imaged.

### Western Blotting for Phospho-p65

Nuclear fraction of the brain was isolated according the previous report (Rajendrasozhan et al., 2010). Total 20 μg of nuclear protein was separated on SDS-PAGE and transferred to polyvinylidene fluoride membranes. The membranes were blocked with 3% BSA for 1 h and incubated overnight with the primary antibodies against phosphor-p65 (#3033, Cell Signaling, Danvers, MA, United States) and lamin B1 (#ab16048, Abcam, Cambridge, MA, United States) at a 1:1,000 dilution in the blocking buffer. After being washed with TBST buffer (20 mM Tris-HCl, pH 7.4, 150 mM NaCl and 0.1% Tween-20) three times for 5 min each, membranes were incubated with the anti-rabbit antibody (#401315, Calbiochem, Gibbstown, NJ, United States) at a 1:30,000 dilution for 1 h and visualized by ECL (Millipore, Billerica, MA, United States).

### Statistical Analysis

Values are reported as means ± SEM. Statistical analyses were conducted using Student’s *t*-test or one-way analysis of variance followed by least significant difference *post hoc* tests. Kaplan–Meier survival curves were compared using the log-rank (Mantel–Cox) test. Differences were considered to be statistically significant at *p* < 0.05.

## Results

### NF-κB Activation in the Brain After GAS Subcutaneous Infection

**FIGURE 1:**
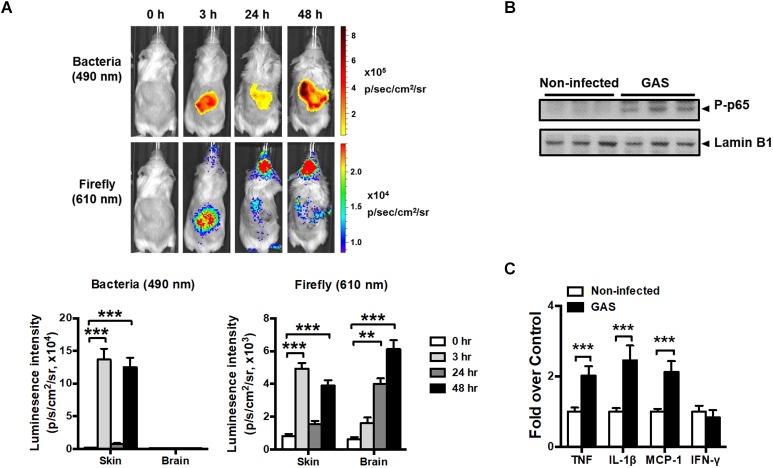
NF-κB activation in the brain after GAS subcutaneous infection. **(A)** Time-scale detection of bacterial and firefly luciferase in the same NF-κB reporter mouse was monitored with spectrum peaks at 490 and 610 nm, respectively, after GAS subcutaneous infection. The photon signals in the back site and brain were quantified using the Living Image software. *n* = 10 mice. **(B)** p65 phosphorylation in the brain was determined using immunoblotting after 48 h post infection (hpi). **(C)** The mRNA levels of TNF, IL-1β, MCP-1, and IFN-γ in the brain were determined using real-time PCR after 48 hpi. *n* = 5–8 mice per group. *^∗∗^p* < 0.01, *^∗∗∗^p* < 0.001.

Transcription factor NF-κB signaling is considered to play a critical role in inflammation and innate immunity. To concurrently monitor the burden of GAS and host innate immune response in the same mouse, the reporter mice carrying firefly luciferase under transcriptional control of the NF-κB promoter were subcutaneously inoculated in the air pouch at the back site with a lethal dose of A20 carrying a *lux* operon and monitored using Living Image software at 3, 24, and 48 h post infection (hpi). Dual bioluminescent signals of bacterial and firefly luciferase in the same mouse were monitored with spectrum peaks at 490 and 610 nm, respectively. The bacterial signal was restricted at the back site of inoculation and decreased at 24 hpi, but reinforced at 48 hpi (Figure 1A, upper panel). Similarly, the firefly luciferase signal rapidly increased at 3 hpi and decreased at 24 hpi, but reinforced at 48 hpi at the inoculation site (Figure 1A, upper panel). Notably, in addition to the infection site, the brain region exhibited firefly luciferase signals, which increased exponentially in a time-dependent manner; however, the bacterial signal in the brain was negative (Figure 1A, upper panel). The dual bioluminescent signals at the back site and brain were quantified (Figure 1A, lower panel). Using non-luminescent GAS can also induce central nervous system inflammation (data not shown).

To confirm the phenomenon in the brain resulting from NF-κB activation, p65 phosphorylation and the expression of NF-κB-mediated downstream genes were detected. We observed that p65 phosphorylation (Figure 1B) and TNF, interleukin (IL)-1β, and monocyte chemoattractant protein (MCP)-1 mRNA expression levels (Figure 1C) were increased in the brain at 48 hpi, but not interferon gamma (IFN-γ) levels, indicating that NF-κB signaling was turned on and triggered neuroinflammation.

### Increased Brain Injury and Microglia Activation After GAS Subcutaneous Infection

**FIGURE 2:**
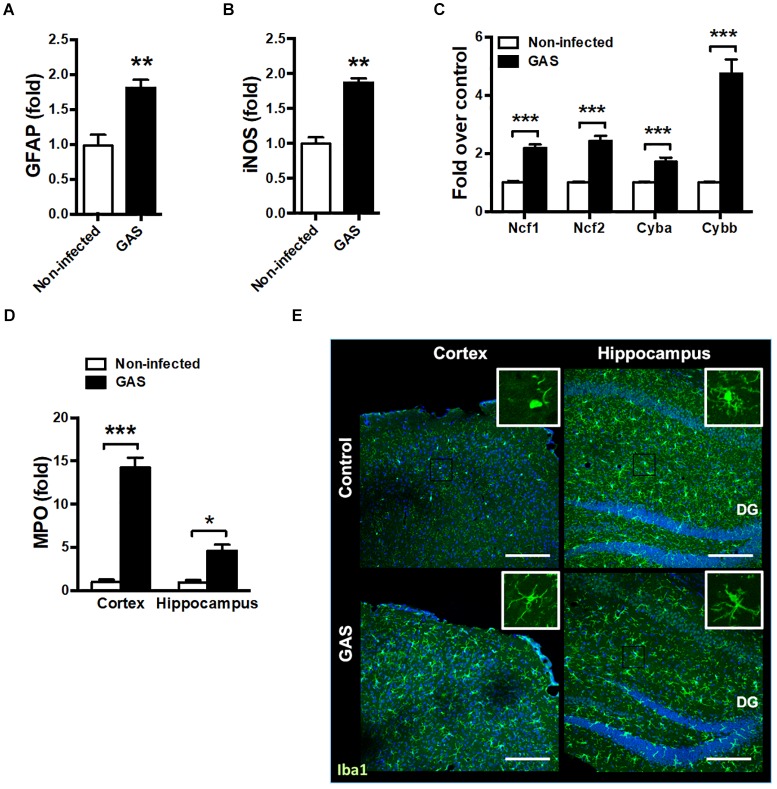
Increased brain injury and microglia activation after GAS subcutaneous infection. Mice were subcutaneously infected with GAS for 48 h. The mRNA levels of GFAP **(A)**, iNOS **(B)**, NADPH oxidase components (Ncf1, Ncf2, Cyba and Cybb) **(C)** in the brain and MPO **(D)** in the cortex and hippocampus were measured using real-time PCR. *n* = 5–6 mice per group. **(E)** Immunofluorescent staining of Iba1 (*green*) in the cortex and hippocampus. The Hoechst nuclear counterstain appears *blue*. Scale bar, 200 μm. ^∗^*p* < 0.05, *^∗∗^p* < 0.01, *^∗∗∗^p* < 0.001.

Because the time point of 48 hpi exhibited the strongest firefly luciferase signal, we examined whether any brain injury markers were detectable during this time point. We found that the inflamed brain abundantly expressed glial fibrillary acidic protein (GFAP), a specific predictor of brain damage (Figure 2A). The inflamed brain overexpressed inducible nitric oxide synthase (iNOS) (Figure 2B) and nicotinamide adenine dinucleotide phosphate (NADPH) oxidase components (Figure 2C), suggesting increased ROS production. Myeloperoxidase (MPO), mainly released by activated macrophages and neutrophils, was abundantly expressed in the cortex and hippocampus of infected mice (Figure 2D). Microglia are specialized innate immune cells in the CNS, which can perform proinflammatory effector functions on activation. Accumulating evidence indicates that microglia activation contributes to neuroinflammation in neurodegenerative diseases (Chen et al., 2016; Carniglia et al., 2017). Therefore, we examined the activation status of microglia at the 48 hpi time point. Immunostaining of brain sections for ionized calcium binding adaptor molecule 1 (Iba1), a marker for microglia, was used for detecting the activation of microglia. Infected mice exhibited activated microglial phenotypes with thicker and shorter processes in the cortex and hippocampus (Figure 2E). These results suggested that subcutaneous GAS infection promotes microglia activation and induces brain injury.

### BBB Leakage Occurred After GAS Subcutaneous Infection

**FIGURE 3:**
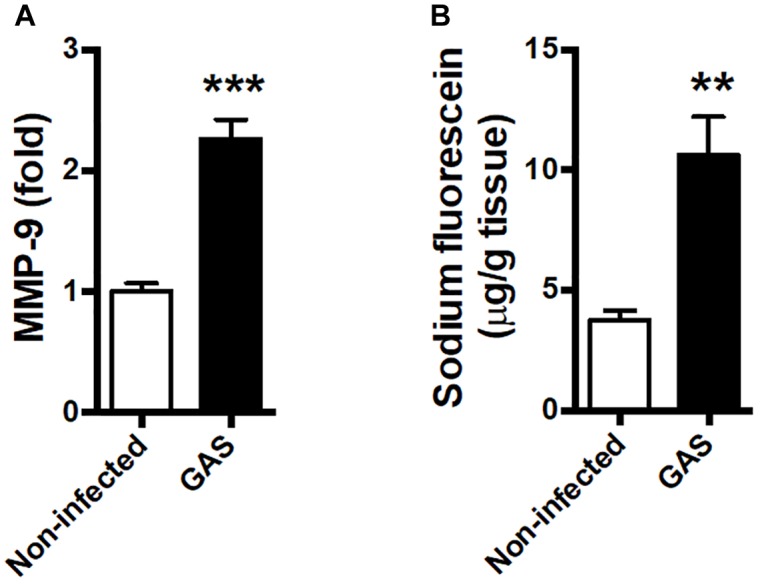
Blood–brain barrier leakage occurred after GAS subcutaneous infection. Mice were subcutaneously infected with GAS for 48 h. **(A)** The mRNA levels of MMP-9 in the brain were determined using real-time PCR. *n* = 5–6 mice per group. **(B)** For BBB permeability detection, sodium fluorescein was intraperitoneally injected. The brain was removed after 40 min and evaluated for fluorescein incorporation. Data are expressed as μg sodium fluorescein per g brain protein. *n* = 4 mice per group. ^∗^*^∗^p* < 0.01, *^∗∗∗^p* < 0.001.

Blood–brain barrier is an important barrier to maintain brain homeostasis by blocking the entrance of toxins and pathogens (Abbott et al., 2010; Wong et al., 2013). Disruption of the BBB is believed to be an early and significant event in neuroinflammation (Banks et al., 2015; Varatharaj and Galea, 2017). We detected an increased matrix metallopeptidase (MMP)-9 expression level in the infected brain (Figure 3A); this is a critical molecule contributed to BBB disruption associated with neuroinflammation (Takata et al., 2011; Dal-Pizzol et al., 2013). Moreover, the permeability of the BBB to small molecules by quantitatively using intraperitoneal sodium fluorescein injection during GAS infection was significantly increased compared with the control group at 48 hpi (Figure 3B).

### Neuronal Degeneration After GAS Subcutaneous Infection

**FIGURE 4:**
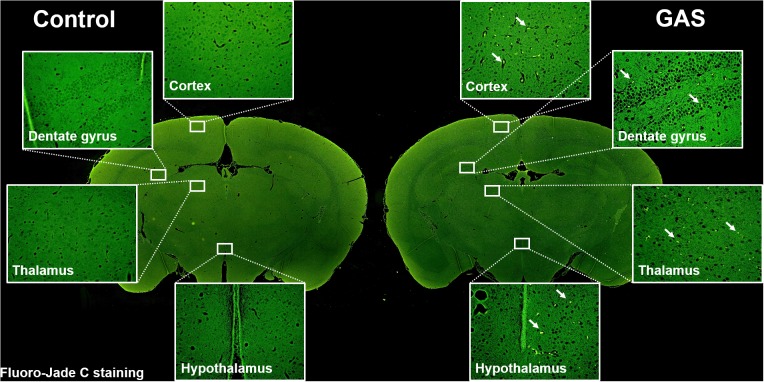
Neuronal degeneration after GAS subcutaneous infection. Mice were subcutaneously infected with GAS for 72 h. Fluoro-Jade C positive cells (*green*) were detected in the whole brain of infected mice.

Sepsis-induced neuroinflammation has been reported to further lead to serious neuronal degeneration (Yokoo et al., 2012). To examine whether the inflamed brain with increased brain injury and BBB permeability finally causes brain pathological change, we performed Fluoro-Jade C staining to analyze neuronal degeneration. Fluoro-Jade C positive cells were identified in each different regions of the brain examined (Figure 4).

### Systemic Inflammation and Elevated TNF Receptor Expression in the Brain

**FIGURE 5:**
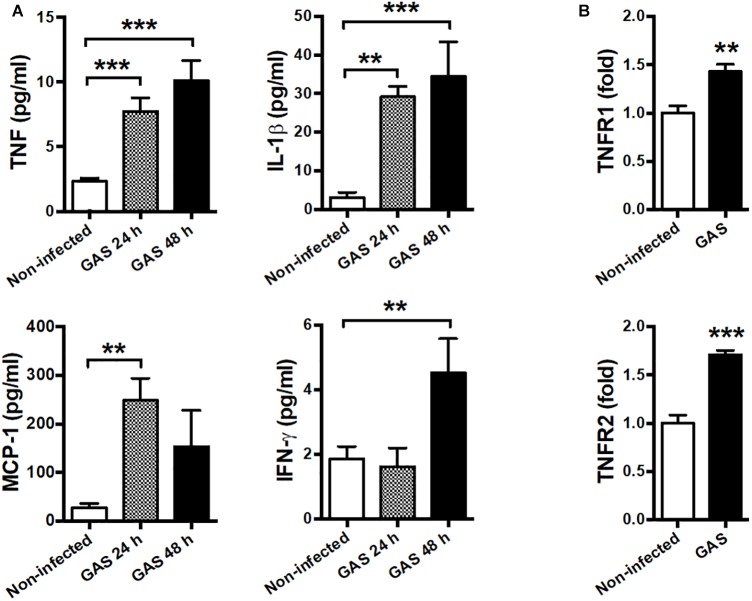
Systemic inflammation and elevated TNF receptor expression in the brain after GAS subcutaneous infection. **(A)** Sera collected from each animal were used for detecting TNF, IL-1β, MCP-1, and IFN-γ levels at 0, 24, and 48 h post infection. *n* = 8–12 mice per group **(B)** After 48 h infection, the mRNA levels of TNFR1 and TNFR2 in the brain were determined using real-time PCR. *n* = 5–8 mice per group. *^∗∗^p* < 0.01, *^∗∗∗^p* < 0.001.

Our infection model demonstrated that circulating cytokines TNF, IL-1β, MCP-1, and IFN-γ were largely increased at both 24 and 48 hpi as a severe systemic inflammatory response (Figure 5A). Therefore, we suggested that neuroinflammation in GAS-infected mice was mediated by the peripheral effect. Peripheral TNF, the key mediator of sepsis, is an acute phase response that initiates a cascade of cytokines. Previous studies have demonstrated that peripheral TNF plays an important role in the pathogenesis of SAE and is involved in the disruption of the BBB in many brain diseases (Sharief and Thompson, 1992; Pan and Kastin, 2007; Alexander et al., 2008; Lv et al., 2010). We observed that TNFR1 and TNFR2 mRNA levels were increased in the brains of infected mice (Figure 5B). Thus, we hypothesized that peripheral TNF is an important mediator of GAS-induced central nervous system inflammation through TNFR signaling.

### Dominant-Negative Inhibitor of TNF (DN-TNF) Attenuates Brain Inflammation

**FIGURE 6:**
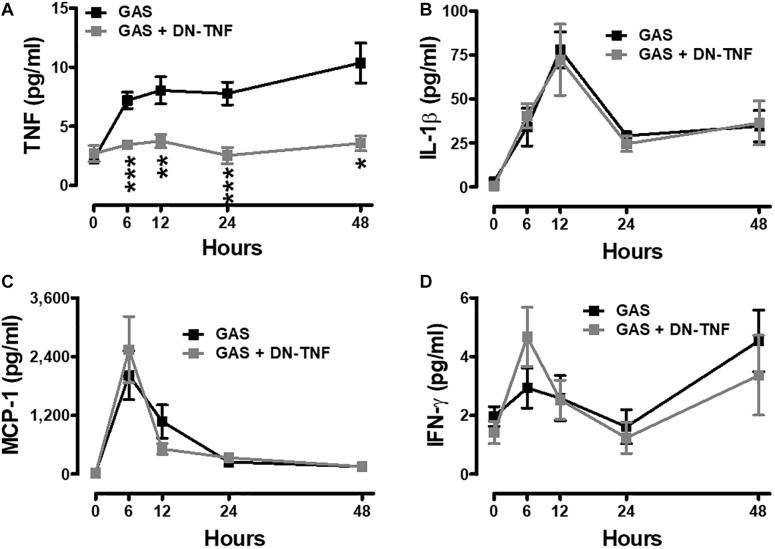
Increased systemic TNF was blocked after GAS subcutaneous infection with DN-TNF treatment. Sera collected from each animal were used for detecting TNF **(A)**, IL-1β **(B)**, MCP-1 **(C)**, and IFN-γ **(D)** levels at 0, 6, 12, 24, and 48 h post infection with three dosages of 50 mg/kg DN-TNF intraperitoneal administration (30 min before infection, 3 h post infection, and 6 h post infection). *n* = 7–10 mice per group. *^∗^p* < 0.05, *^∗∗^p* < 0.01, *^∗∗∗^p* < 0.001.

**FIGURE 7:**
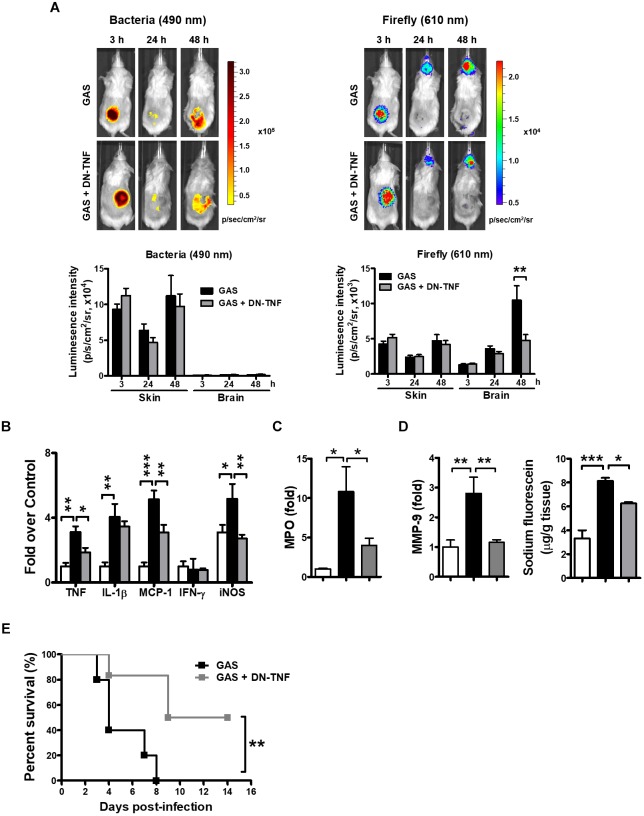
Amelioration of GAS-induced brain inflammation by blocking circulating TNF. **(A)** During the infection period, NF-κB reporter mice were intraperitoneally administered three dosages of 50 mg/kg DN-TNF, and then time-scale detection of bacterial and firefly luciferase in the same mouse was monitored, with spectrum peaks at 490 and 610 nm, respectively. The photon signals in the back site and brain were quantified using Living Image software. *n* = 10 mice per group. The mRNA levels of inflammatory cytokines **(B)**, MPO **(C)**, and MMP-9 **(D)** in the brain were determined using real-time PCR after 48 hpi. *n* = 4–6 mice per group. **(D)** For BBB permeability detection, sodium fluorescein was intraperitoneally injected. *n* = 4–5 mice per group. **(E)** Survival of GAS-infected mice with or without DN-TNF treatment. *n* = 5–6 mice per group. *^∗^p* < 0.01, *^∗∗^p* < 0.01, *^∗∗∗^p* < 0.001.

To determine whether circulating TNF blockage can attenuate subcutaneous GAS infection-induced central nervous system inflammation, we treated infected mice with three doses (30 min before, 3 h after infection, and 6 h after infection) of a dominant-negative inhibitor of TNF (DN-TNF) and detected the luciferase signal at 3, 24, and 48 hpi. We first detected the circulating TNF level after DN-TNF treatment and found that DN-TNF can successfully block circulating TNF, but not IL-1β, MCP-1, and IFN-γ levels at 6, 12, 24, and 48 hpi (Figure 6A–D). From the bioluminescence results, the bacterial signal exhibited no difference between the GAS and GAS plus DN-TNF treatment groups (Figure 7A), suggesting that the host defense mechanisms still functioned after DN-TNF treatment. However, the firefly luciferase signal from the infected brain was significantly decreased after DN-TNF treatment, particularly at 48 hpi (Figure 7A). The dual bioluminescent signals at the back site and brain were quantified. DN-TNF treatment reduced the inflammatory cytokine and MPO expression levels in the infected brain (Figure 7B,C). In addition, DN-TNF treatment significantly reduced the MMP-9 expression and sodium fluorescein content in the brain (Figure 7D). Results showed that the infected mice with DN-TNF treatment had a higher survival rate (median survival 11.5 days) than the infected mice without DN-TNF treatment (median survival 4 days) (Figure 7E).

## Discussion

In the past few decades, GAS has been identified as causing various diseases with a wide range of severity. Among them, the most severe is GAS-associated sepsis syndrome, which is associated with a high mortality rate. However, until now, no study has identified whether GAS-associated sepsis syndrome can induce central nervous system inflammation, which is difficult to correctly diagnose because patients are usually sedated, which can mask neurological disturbances. Previously, GAS-induced CNS disorders were believed to be caused by bacteria directly penetrating into the brain or mediated by autoantibodies recognizing CNS epitopes. However, we found the two case reports showing that patients exhibited brain illness without the existence of GAS in the brain (Maytan, 2000; Yanagawa et al., 2009). Furthermore, in these two cases the disease progressions were too short to induce autoantibody production, suggesting these CNS disorders were not like PANDAS. We examined pathological changes in the brain in a mouse model of GAS subcutaneous infection. The infected mice exhibited neuroinflammation and microglial activation accompanied with brain injury and BBB damage. The present study evidenced the existence of subcutaneous GAS-induced central nervous system inflammation.

Although it is known that severe GAS infection can induce septic shock and an increased risk of fatal outcome, no animal study has further investigated GAS-induced central nervous system inflammation. Unlike traditional methods of investigating bacterial pathogenesis and host immune responses in a mouse infection model by euthanizing groups of animals at multiple time points, *in vivo* bioluminescence imaging techniques prevent variations in individual mice and enable the identification of unexpected sites of host response that might be missed when specific tissues are analyzed at predetermined time points. Moreover, the use of bioluminescent bacteria *in vivo* allows longitudinal studies of bacteria replication and dissemination in mice. We selected NF-κB reporter mice as a specific readout for proinflammatory responses and bioluminescent GAS as infection bacteria to monitor the inflammation sites and track bacterial dissemination. Notably, excepting the infection site, the bioluminescent signal in the brain of the reporter mice was detected from NF-κB activation not the bacterial signal. Considering the detection limit of bacterial signal, no evidence was obtained to exclude the possibility of GAS direct translocation to the brain. Circulating IFN-γ is crucial for immunity against invading pathogens through the activating innate immune responses of macrophages. It is interesting to find that the expression level of IFN-γ was increased in the circulation, but not in the brain of infected mice. Here, we speculated that this may be due to the expression of IFN-γ is not through NF-κB signaling in the brain, therefore there is no increased IFN-γ in the brain.

In our study, we supposed that the neuroinflammation was mediated by secondary infection—systemic inflammation. TNF is a cytokine produced in the early phase of inflammation and plays a role in immune-to-brain communication by enhancing the leakiness of the BBB (Mayhan, 2002) and further promoting microglia activation (Kuno et al., 2005). It seems that anti-TNF therapy could improve GAS-mediated neuroinflammation through reducing BBB leakage and microglia activation. However, previous studies in GAS-infected mouse model have demonstrated that the effect of anti-TNF treatment is controversial (Martin et al., 1993; Wayte et al., 1993; Stevens et al., 1996; Renaud et al., 2011). The infection model used and the TNF targeting method and efficiency are factors causing varied results. To mimic the human infected model, we selected a mouse infection model of subcutaneous GAS injection and detected the pathogenic changes in the brain. Regarding the anti-TNF strategy, compared with most non-selective anti-TNF biologics, we used a novel class of dominant-negative TNF biologic, XENP1595, which has been proved to attenuate experimental arthritis, experimental autoimmune encephalomyelitis, and Parkinson disease in animal models, without suppressing the innate immunity to infection (McCoy et al., 2006; Zalevsky et al., 2007; Brambilla et al., 2011). In the GAS infection model, mice with a peripheral treatment of XENP1595 exhibited reduced brain inflammation, microglial activation, and BBB leakage. In addition, the improved survival may be contributed from reduced peripheral TNF and central nervous system inflammation.

Anti-TNF therapy offers a targeted strategy different from that of non-specific immunosuppressive agents and has been considered an option for many chronic inflammatory diseases, such as rheumatoid arthritis, psoriasis, and inflammatory bowel disease. However, the use of anti-TNF therapy is associated with the risk of infections because of the impairment of host immunity. XENP1595 is a TNF blocker that causes selective pharmacological inhibition of soluble TNF (solTNF) without blocking transmembrane TNF (tmTNF), versus other anti-TNFs, including etanercept, infliximab, and adalimumab, blocking both tmTNF and solTNF (Steed et al., 2003; Sedger and McDermott, 2014). Previous reports have demonstrated that XENP1595, the solTNF blocker, attenuated mouse arthritis without suppressing innate immunity to *Listeria* infection and protected mice from acute liver inflammation without suppressing innate immunity to mycobacterial infections, suggesting that tmTNF confers protection to infection (Zalevsky et al., 2007; Olleros et al., 2009). In our study, the infected mice treated with XENP1595 exhibited bacterial load at the inoculation site comparable to that of the vehicle-treated controls. The ability to prevent the bacterial outgrowth suggests that antibacterial response exists in the absence of solTNF and is mediated through tmTNF. We observed that treatment with XENP1595 resulted in a significant reduction of only TNF, but not other inflammatory cytokines such as IL-1β, MCP-1, and IFN-γ, suggesting that even without the effect of TNF, the host immune system can still function to defend against GAS.

Brain inflammation-induced injury is a life-threatening outcome of *S. pneumoniae* meningitis (Xu et al., 2017). Traditional treatment of *S. pneumoniae* infection by antibiotic only reduced bacterial load but not be able to rescue neuronal injury (Kadurugamuwa et al., 2005). BBB opening controlled by TNF is necessary for the development of meningitis (Tsao et al., 2002; Prager et al., 2017). Previous studies showed that an inhibitor of TNF-α converting enzyme and MMP efficiently decreased neuronal injury and improved survival without inhibiting bacterial growth (Meli et al., 2004; Echchannaoui et al., 2007). In our study, DN-TNF exhibited the ability to ameliorate brain inflammation in infected mice but displayed no effect on local bacterial clearance. According to the individual protective effects of DN-TNF and antibiotics, the use of combination of both treatments may be a consummate strategy for life-threatening bacterial infection.

In this study, we used a dual reporter system to monitor the interaction between GAS and host immunity and identified the novel inflammation site that was neglected before. In addition to the infection site, we found that subcutaneous GAS-induced systemic inflammation caused brain inflammation and neuron degeneration. Treatment with the selective inhibitor of solTNF exhibited a therapeutic effect without causing GAS outgrowth. Our findings provide new insight into how GAS subcutaneous infection lead to brain inflammation and suggest potential therapeutic targets.

## Author Contributions

Y-HL, P-HW, C-CK, and P-JT conceived and designed the experiments. Y-HL and P-HW performed most of the experiments. C-CK performed some experiments. Y-HL, P-HW, C-CK, and C-TL analyzed the data. C-KC contributed tools. Y-ST and J-JW participated in discussion. Y-HL and P-JT wrote the paper.

## Conflict of Interest Statement

The authors declare that the research was conducted in the absence of any commercial or financial relationships that could be construed as a potential conflict of interest.
